# Subclinical leaflet thrombosis after transcatheter aortic valve implantation: no association with left ventricular reverse remodeling at 1-year follow-up

**DOI:** 10.1007/s10554-021-02438-2

**Published:** 2021-10-16

**Authors:** Jurrien H. Kuneman, Gurpreet K. Singh, Nicolaj C. Hansson, Laura Fusini, Steen H. Poulsen, Federico Fortuni, E. Mara Vollema, Anders L. D. Pedersen, Andrea D. Annoni, Bjarne L. Nørgaard, Gianluca Pontone, Nina Ajmone Marsan, Victoria Delgado, Jeroen J. Bax, Juhani Knuuti

**Affiliations:** 1 Department of Cardiology, Heart Lung Center, Leiden University Medical Center, Albinusdreef 2, 2300 RC Leiden, The Netherlands; 2 Department of Cardiology, Aarhus University Hospital, Aarhus, Denmark; 3 Department of Cardiovascular Imaging, Centro Cardiologico Monzino IRCCS, Milan, Italy; 4 Department of Cardiology, Azienda Ospedaliero Universitaria Città della Salute e della Scienza di Torino, Turin, Italy; 5 Turku PET Centre, University of Turku and Turku University Hospital, Turku, Finland

**Keywords:** Transcatheter aortic valve implantation, Subclinical leaflet thrombosis, Hypo-attenuated leaflet thickening, Multi-detector row computed tomography, Left ventricular reverse remodeling, Aortic stenosis

## Abstract

Hypo-attenuated leaflet thickening (HALT) of transcatheter aortic valves is detected on multidetector computed tomography (MDCT) and reflects leaflet thrombosis. Whether HALT affects left ventricular (LV) reverse remodeling, a favorable effect of LV afterload reduction after transcatheter aortic valve implantation (TAVI) is unknown. The aim of this study was to examine the association of HALT after TAVI with LV reverse remodeling. In this multicenter case–control study, patients with HALT on MDCT were identified, and patients without HALT were propensity matched for valve type and size, LV ejection fraction (LVEF), sex, age and time of scan. LV dimensions and function were assessed by transthoracic echocardiography before and 12 months after TAVI. Clinical outcomes (stroke or transient ischemic attack, heart failure hospitalization, new-onset atrial fibrillation, all-cause mortality) were recorded. 106 patients (age 81 ± 7 years, 55% male) with MDCT performed 37 days [IQR 32–52] after TAVI were analyzed (53 patients with HALT and 53 matched controls). Before TAVI, all echocardiographic parameters were similar between the groups. At 12 months follow-up, patients with and without HALT showed a significant reduction in LV end-diastolic volume, LV end-systolic volume and LV mass index (from 125 ± 37 to 105 ± 46 g/m^2^, p = 0.001 and from 127 ± 35 to 101 ± 27 g/m^2^, p < 0.001, respectively, p for interaction = 0.48). Moreover, LVEF improved significantly in both groups. In addition, clinical outcomes were not statistically different. Improvement in LVEF and LV reverse remodeling at 12 months after TAVI were not limited by HALT.

## Introduction

Pressure overload of the left ventricle (LV) caused by severe aortic valve stenosis commonly leads to LV remodeling and LV hypertrophy [1, 2]. If left untreated, this is associated with a significantly increased risk of morbidity and mortality [3, 4]. Aortic valve replacement provides direct relief of the LV outflow obstruction. Subsequently, the myocardium may undergo a favorable process of LV reverse remodeling with reduction in LV volumes, regression of LV mass, and improvement in function [5–8]. LV mass regression after aortic valve replacement has been associated with improved survival [9, 10]. Moreover, data from the Placement of Aortic Transcatheter Valves (PARTNER) trial demonstrated that greater LV mass regression after transcatheter aortic valve implantation (TAVI) was associated with reduced heart failure-related hospitalizations during 1 year follow-up [11].

Hypo-attenuated leaflet thickening (HALT) of transcatheter aortic valves can be observed on multidetector-row computed tomography (MDCT) and is considered as an early marker of leaflet thrombosis [12–17]. The incidence of HALT varies between 4 and 40% [12–15, 18–21]. Additionally, previous data suggested that valve thrombosis is considered to have a significant impact on hemodynamic prosthetic valve deterioration [22], which has been linked with less LV reverse remodeling after aortic valve replacement [23]. However, whether HALT affects LV reverse remodeling after TAVI is unknown. Accordingly, the potential relation between HALT, prosthetic valve gradients, and LV reverse remodeling was evaluated, as well as the relation between HALT and clinical events after TAVI.

## Methods

### Patient population

In this multicenter retrospective case–control study, patients who underwent MDCT after TAVI between 2007 and 2019 were evaluated. The study was conducted at three sites: Leiden University Medical Center, Leiden, The Netherlands; Aarhus University Hospital, Aarhus, Denmark, and Centro Cardiologico Monzino IRCCS, Milan, Italy. All patients had undergone a post-procedural contrast-enhanced MDCT scan, 1–3 months after TAVI as per institution protocol to assess prosthetic valve positioning and deployment (in Leiden and Aarhus) or as clinically indicated (Milan). Patients with HALT of the transcatheter heart valve evaluated by MDCT were identified. Thereafter, using propensity score matching, patients without HALT were identified and further matched to the patients with HALT according to valve type and size, baseline LV ejection fraction (LVEF), sex, age, and time of CT scan. Demographic and clinical data were obtained from electronic patient files. This retrospective analysis complies with the Declaration of Helsinki and was approved by the institutional review board which waived the need for written informed consent.

### Transcatheter aortic valve procedure

Eligibility and feasibility of TAVI as well as decision-making on the access route and valve type were at the discretion of the local heart teams. Selection of transcatheter heart valve size was based on MDCT measurements of the aortic annulus, as previously described [13, 24]. The TAVI procedure was performed according to standard practice [25]. Balloon- and self-expandable valves were used: Edwards SAPIEN, SAPIEN XT, SAPIEN 3 (Edwards Lifesciences, Irvine, CA, USA), Medtronic CoreValve Evolut (Medtronic, MN, Minnesota, USA), and Boston Scientific Lotus Edge (Boston Scientific, Natick, Massachusetts, USA). After TAVI, all patients received dual antiplatelet therapy for 3 to 12 months and thereafter lifelong monotherapy with aspirin or clopidogrel. If oral anticoagulants were indicated, the decision for additional treatment with antiplatelet therapy was left at the discretion of the treating cardiologist taking into consideration the bleeding risks.

### Echocardiographic follow-up

Transthoracic echocardiographic examinations were performed before TAVI, immediately post-TAVI (pre-discharge), and at 12 months follow-up. LV function and dimensions (LV end-diastolic diameter, intraventricular septum thickness, and LV posterior wall thickness) were assessed before and 12 months after TAVI. Prosthetic valve hemodynamics (valve area, transvalvular gradient) were assessed immediately after TAVI and at 12 months follow-up. All echocardiographic examinations were acquired by experienced echocardiographers using Vivid-7, Vivid E9 (General Electric Vingmed, Horten, Norway), iE33, or EPIQ (Philips Medical Systems, Best, Netherlands) ultrasound systems. Prosthetic valve hemodynamics, as well as LV function and dimensions, were reported according to current guidelines [26, 27]. Peak and mean transvalvular gradients were calculated from continuous wave Doppler recordings of the apical 3- or 5-chamber views according to the Bernoulli equation. Prosthetic aortic valve area (AVA) was calculated using the continuity equation. LV volumes (end-diastolic and end-systolic) were assessed using planimetry based on apical 2- and 4-chamber views and were indexed to body surface area. LVEF was estimated using Simpson’s biplane method. LV dimensions were obtained in the parasternal long-axis view at end-diastole [26]. LV mass was calculated using the Devereux formula and was indexed to body surface area [26].

### MDCT image acquisition and analysis

**Fig. 1:**
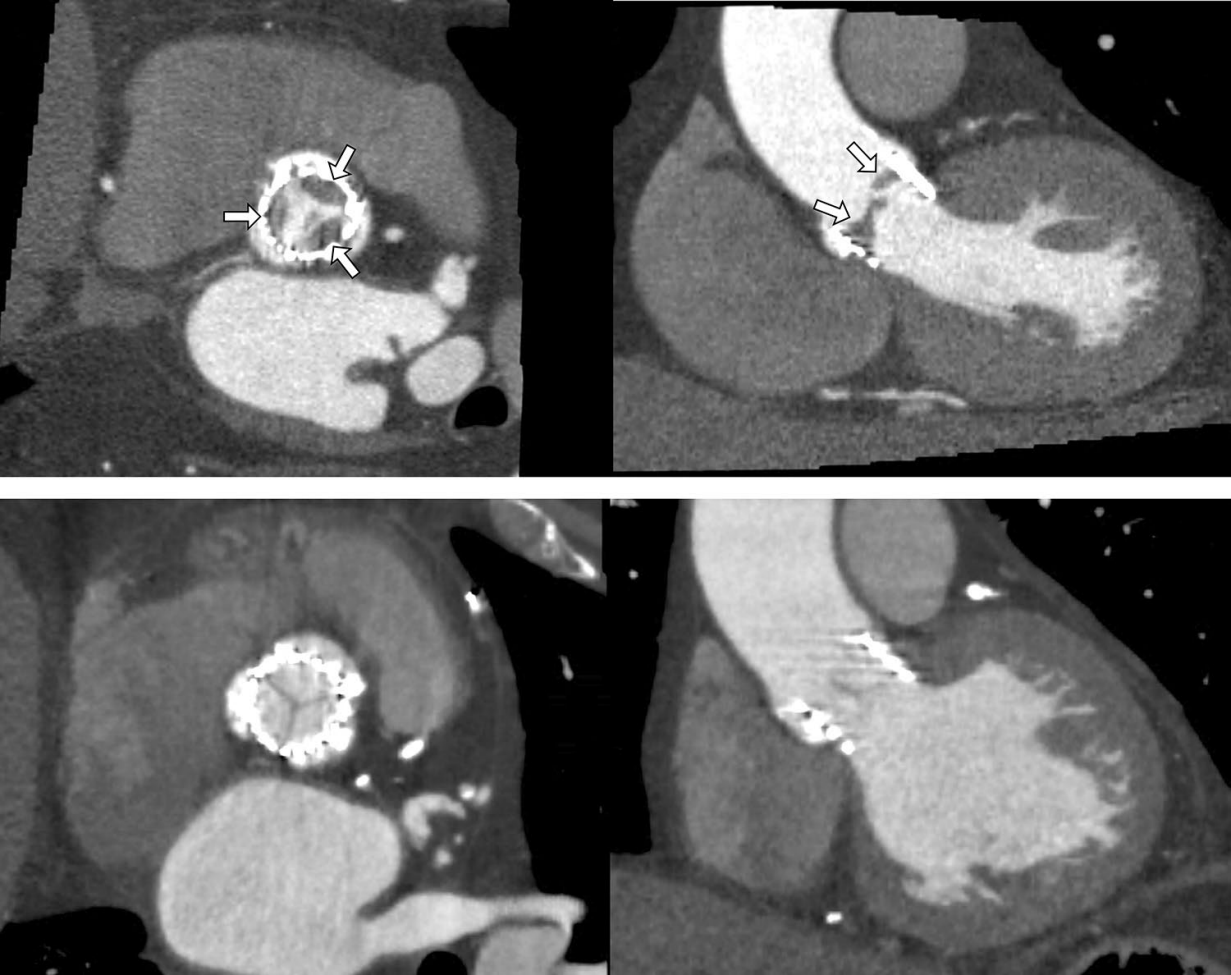
Multiplanar reconstructions of transcatheter aortic valves on multidetector row computed tomography with HALT (white arrows, upper panel) and without HALT (lower panel)

Post-procedural contrast-enhanced MDCT scans were performed using a 64-row (Aquilion 64, Toshiba Medical Systems, Tochigi-ken, Japan), 256-row (Revolution CT, GE Healthcare, Chicago, IL, USA), 320-row (AquilionOne; Toshiba Medical Systems) or second-generation dual-source (Siemens Somatom Definition Flash, Siemens Healthcare, Erlangen, Germany) computed tomography scanners depending local equipment. Methods for image acquisition have been reported previously [13, 15, 28]. Image analysis was performed using dedicated software (Vitrea FX 6.5; Vital Images, Minnetonka, MN, USA; ADW 4.7, GE Healthcare or Multimodality Workplace, Siemens Healthcare). Post-TAVI MDCT scans were used to assess the presence of HALT. HALT was defined as a hypo-attenuated abnormality attached to the valve affecting one or more leaflets and was assessed by 2-dimensional multiplanar reformation planes (Fig. 1), as described previously [13, 15, 24].

### Endpoints

The echocardiographic endpoints included (a) abnormal valve hemodynamics indicating significant stenosis defined as a mean gradient of the prosthetic valve ≥ 20 mmHg and a valve area of ≤ 1.1 cm ^2^ [27], (b) decrease in LVEF ≥ 5%, (c) no reduction in LV volumes or (d) no reduction in LV mass. LV reverse remodeling was defined as a reduction in LV end-diastolic volume, LV end-systolic volume, or regression in LV mass at 12 months after TAVI compared to baseline. In addition, clinical outcomes included the occurrence of stroke or transient ischemic attack (TIA), heart failure hospitalization, new-onset atrial fibrillation, and all-cause mortality during the follow-up.

### Statistical analysis

Distribution of continuous variables was evaluated using histograms and Q-Q plots. Continuous variables following a normal distribution are presented as mean ± standard deviation (SD) and were compared using the independent Student *t* test. Non-normally distributed variables are presented as median with 25–75% interquartile range (IQR) and were compared using the Mann–Whitney *U* test. Categorical variables are presented as absolute values and percentages and were compared using the χ^2^ test or Fisher’s exact test as appropriate. General linear models were used to evaluate changes in LV volumes and mass as well as LVEF and prosthetic valve hemodynamics between patients with versus without HALT over time. The Greenhouse–Geisser correction was used if the sphericity assumption was violated. Additional analyses were performed to correct for the potential confounding effect of age, sex, hypertension, diabetes, coronary artery disease, previous myocardial infarction, and pre-TAVI LVEF on LV reverse remodeling, as well as for oral anticoagulation treatment on HALT, and were used as covariates in the general linear models. Kaplan–Meier curves were generated to estimate the cumulative survival rates of clinical outcomes and the log-rank test was used to compare differences between patients with versus without HALT. Twenty random individuals were selected for the evaluation of intra- and inter-observer variability for the presence of HALT and were performed using Cohen’s κ test. A strong agreement was defined by a Cohen’s κ > 0.80. A two-sided p value < 0.05 was considered significant. Data analysis was performed with SPSS version 25.0 (IBM SPSS Statistics, IBM Corporation, Armonk, New York, USA).

## Results

### Patients and procedural characteristics

A total of 106 patients (mean age 81 ± 7 years, 55% male) were included in this analysis, comprising 53 patients with HALT and 53 matched controls. MDCT was performed 37 days (IQR 32–76) after TAVI. The intra- and inter-observer variability for the presence of HALT demonstrated a strong agreement (Cohen’s κ = 0.92 for both). Patient characteristics of the total population and comparison between patients with and without HALT are summarized in Table 1. Patients with HALT had more frequently a history of stroke or TIA before TAVI (p = 0.038). Other clinical baseline characteristics were similar between the groups. Overall, oral anticoagulation was used in 29% of patients and oral anticoagulation plus antiplatelet therapy in 15% of patients. Interestingly, the antithrombotic regimen did not differ between patients with and without HALT before TAVI.

**Table 1:** Baseline clinical characteristics

Variable	Overall population (n = 106)	HALT (n = 53)	no HALT (n = 53)	p value
Age, years	81 ± 7	81 ± 7	80 ± 7	0.47
Male, n (%)	58 (55)	31 (59)	27 (51)	0.44
Body surface area, m^2^	1.84 ± 0.20	1.86 ± 0.22	1.82 ± 0.17	0.29
Body mass index, kg/m^2^	27 ± 4	27 ± 4	26 ± 4	0.87
EUROSCORE	12.3 (7.4–20.6)	13.0 (7.5–21.4)	11.7 (6.4–19.7)	0.34
Creatinine level, µmol/ml	88 (75–108)	87 (75–105)	88 (74–111)	0.70
Hypertension, n (%)	74 (70)	37 (70)	37 (70)	> 0.99
Hypercholesterolemia, n (%)	71 (67)	37 (70)	34 (64)	0.54
Diabetes, n (%)	27 (26)	11 (21)	16 (30)	0.27
Previous or current smoking, n (%)	14 (38)	5 (28)	9 (47)	0.22
CAD, n (%)	49 (46)	26 (49)	23 (43)	0.56
Previous revascularization, n (%)				
PCI	23 (22)	9 (17)	14 (26)	0.23
CABG	20 (19)	13 (25)	7 (13)	
NYHA classification, n (%)				
I–II	41 (39)	18 (34)	23 (43)	0.32
III–IV	65 (61)	35 (66)	30 (56)	
Previous myocardial infarction, n (%)	13 (12)	6 (11)	7 (13)	0.77
Previous stroke/TIA, n (%)	13 (12)	10 (19)	3 (6)	0.038
Peripheral vascular disease, n (%)	24 (23)	11 (21)	13 (25)	0.64
Chronic obstructive pulmonary disease, n (%)	19 (18)	10 (19)	9 (17)	0.80
Atrial fibrillation, n (%)	23 (22)	13 (25)	10 (19)	0.48
Medication, n (%)				
Beta-blocker	56 (53)	29 (55)	27 (51)	0.70
ACE-I/ARB	67 (63)	35 (66)	32 (60)	0.55
Calcium antagonist	30 (28)	14 (26)	16 (30)	0.67
Diuretics	64 (60)	33 (62)	31 (59)	0.69
Spironolactone	22 (21)	11 (21)	11 (21)	> 0.99
Statins	54 (51)	25 (47)	29 (55)	0.44
Antiplatelet	89 (84)	44 (83)	45 (85)	0.79
Anticoagulation	31 (29)	13 (25)	18 (34)	0.29
Anticoagulation + antiplatelet therapy	16 (15)	5 (9)	11 (21)	0.10

Data are presented as mean ± SD, median (IQR) and n (%)

*ACE-I* angiotensin-converting enzyme, *ARB II* angiotensin II receptor blocker, *CABG* coronary artery bypass grafting, *CAD* coronary artery disease, *NYHA* New York Heart Association, *PCI* percutaneous coronary intervention, *TIA* transient ischemic attack

Baseline echocardiographic characteristics are summarized in Table 2. The mean LVEF was 52 ± 12% before TAVI and the mean LV mass index was 126 ± 36 g/m^2^ in the overall population. All baseline echocardiographic parameters were comparable between patients with and without HALT. Bicuspid aortic valves were present in 5% of the population.

**Table 2:** Baseline (pre-TAVI) echocardiographic data

Variable	Overall population (n = 106)	HALT (n = 53)	no HALT (n = 53)	p value
Bicuspid aortic valve, n (%)	5 (5)	3 (6)	2 (4)	0.67
LVEF, %	52 ± 12	53 ± 11	51 ± 13	0.35
LVEDV, ml	95 ± 32	93 ± 32	98 ± 33	0.51
LVESV, ml	47 ± 23	44 ± 20	50 ± 26	0.20
LVEDVi, ml/m^2^	52 ± 17	50 ± 16	54 ± 17	0.30
LVESVi, ml/m^2^	26 ± 12	24 ± 10	27 ± 14	0.12
SV, ml	49 ± 17	49 ± 18	48 ± 17	0.62
SVi, ml/m^2^	27 ± 9	27 ± 10	26 ± 9	0.81
LV mass, g	231 ± 70	232 ± 75	230 ± 66	0.89
LV mass index, g/m^2^	126 ± 36	125 ± 37	127 ± 35	0.76

Data are presented as mean ± SD and n (%)

*LVEDV* left ventricular end-diastolic volume, *LVEDV* left ventricular end-diastolic volume index, *LVEF* left ventricular ejection fraction, *LVESV* left ventricular end-systolic volume, *LVESVi* left ventricular end-systolic volume index, *SV* stroke volume, *SVi* stroke volume index, *TAVI* transcatheter aortic valve implantation

The majority of the TAVI procedures were performed in native aortic valves but in 7 patients (7%) a valve-in-valve procedure was performed. Procedure access was transfemoral in 85 patients (80%), transapical in 20 patients (19%), and transaortic access in one patient. The majority of patients received balloon-expandable valves: Edwards SAPIEN 3 (64%), SAPIEN XT (13%), and SAPIEN (14%). Self-expandable valves as the Medtronic Corevalve Evolut was used in six patients (6%) and the Boston Scientific Lotus in 3%. Prosthesis size ranged from 20 to 31 mm, with 26 mm being most frequently used in 47 patients (44%).

### Echocardiographic results after TAVI

**Fig. 2:**
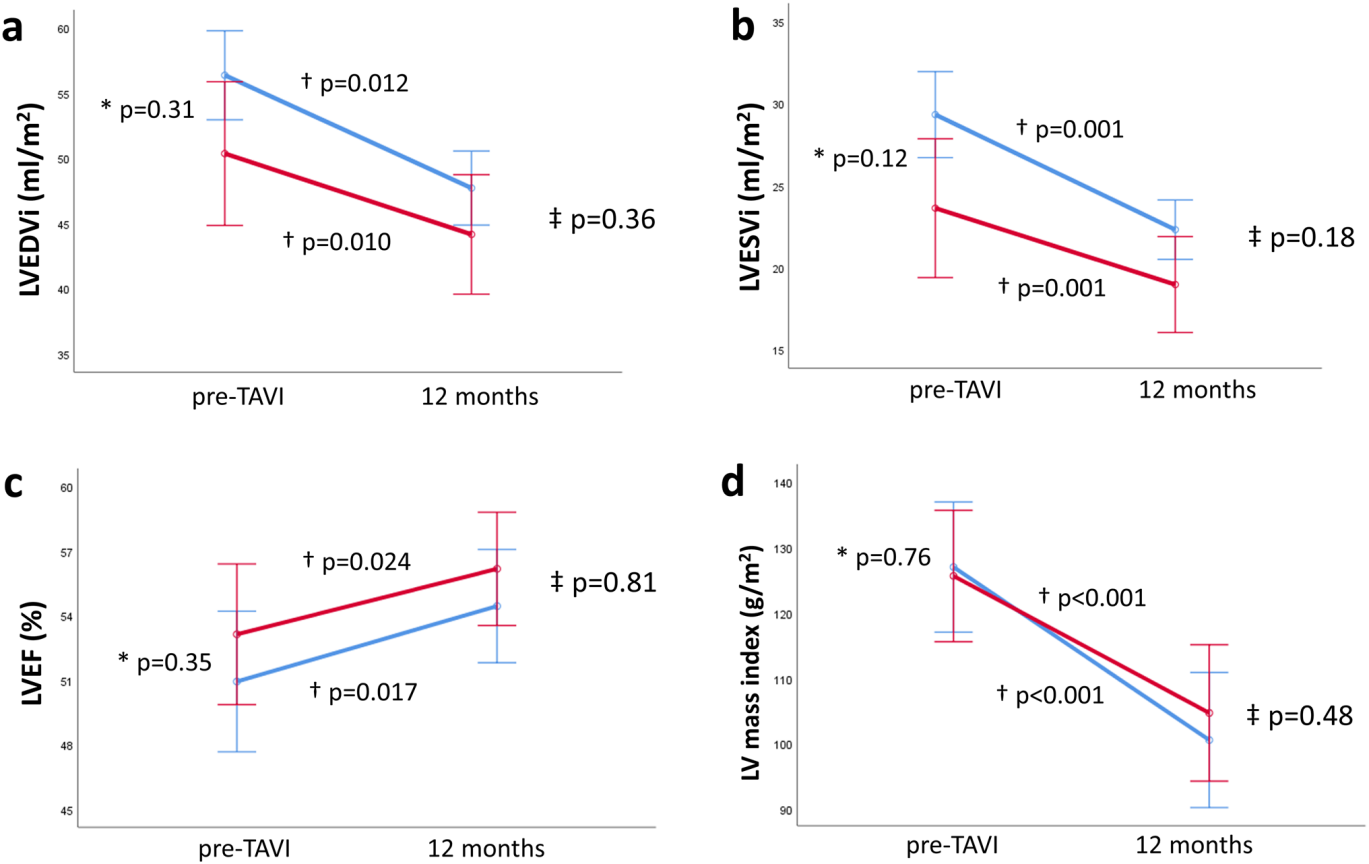
Left ventricular reverse remodeling in patients with (red line) and without hypo-attenuated leaflet thickening (blue line) after transcatheter aortic valve implantation. Changes in left ventricular end-diastolic volume index (LVEDVi, **a**), left ventricular end-systolic volume index (LVESVi, **b**), left ventricular ejection fraction (LVEF, **c**) and left ventricular mass index (**d**) from baseline to 12 months follow-up after transcatheter aortic valve implantation. *shows p value for comparing means between groups at baseline. †shows p-value for groups over time. ‡ shows p for interaction between groups over time

Prosthetic valve hemodynamics immediately after TAVI are shown in Table 3. Mean transvalvular gradient in the total population was 11.7 ± 6.0 mmHg and the prosthetic aortic valve area was 1.64 ± 0.43 cm^2^. At 12 months post-TAVI, both patients with HALT and without HALT showed a significant reduction in LV end-diastolic volume index (LVEDVi, from 50 ± 16 to 44 ± 17 ml/m^2^, p = 0.010 and from 54 ± 17 to 48 ± 14 ml/m^2^, p = 0.012, respectively) and in LV end-systolic volume index (LVESVi, from 24 ± 10 to 19 ± 8 ml/m^2^, p = 0.001 and from 27 ± 14 to 22 ± 9 ml/m^2^, p = 0.001, respectively), without significant differences between the groups over time (p for interaction = 0.36 and p = 0.18, respectively). Additionally, LV mass index regressed significantly in both groups (HALT: from 125 ± 37 to 105 ± 46 g/m^2^, p = 0.001; no HALT: from 127 ± 35 to 101 ± 27 g/m^2^, p < 0.001; p for interaction = 0.48). LVEF improved significantly at 12 months follow-up in both groups of patients, without significant differences between patients with and without HALT over time (HALT: from 53 ± 11 to 56 ± 8%, p = 0.024; no HALT: from 51 ± 13 to 54 ± 11%, p = 0.017; p for interaction = 0.81). Stroke volume index remained unchanged in both groups. Similar results were observed when adjusting for potential confounders of LV reverse remodeling and the potential confounding role of oral anticoagulation treatment on the presence of HALT: no significant differences were noted between patients with and without HALT over time with regards to reduction of LV volumes (LVEDVi: adjusted p for interaction = 0.45; LVESVi: p = 0.65) and LV mass index (adjusted p for interaction = 0.86) as well as improvement in LVEF (adjusted p for interaction = 0.39). Figure 2 presents the changes in LV variables between patients with and without HALT over time.

**Table 3:** Prosthetic valve hemodynamics immediately after TAVI

	Overall population (n = 106)	HALT (n = 53)	no HALT (n = 53)	p value
Peak transvalvular gradient, mmHg	22.2 ± 10.7	21.1 ± 10.2	23.3 ± 11.3	0.30
Mean transvalvular gradient, mmHg	11.7 ± 6.0	11.4 ± 6.2	12.1 ± 5.9	0.60
Aortic valve area, cm^2^	1.64 ± 0.43	1.61 ± 0.42	1.67 ± 0.44	0.61

*TAVI* transcatheter aortic valve implantation

**Fig. 3:**
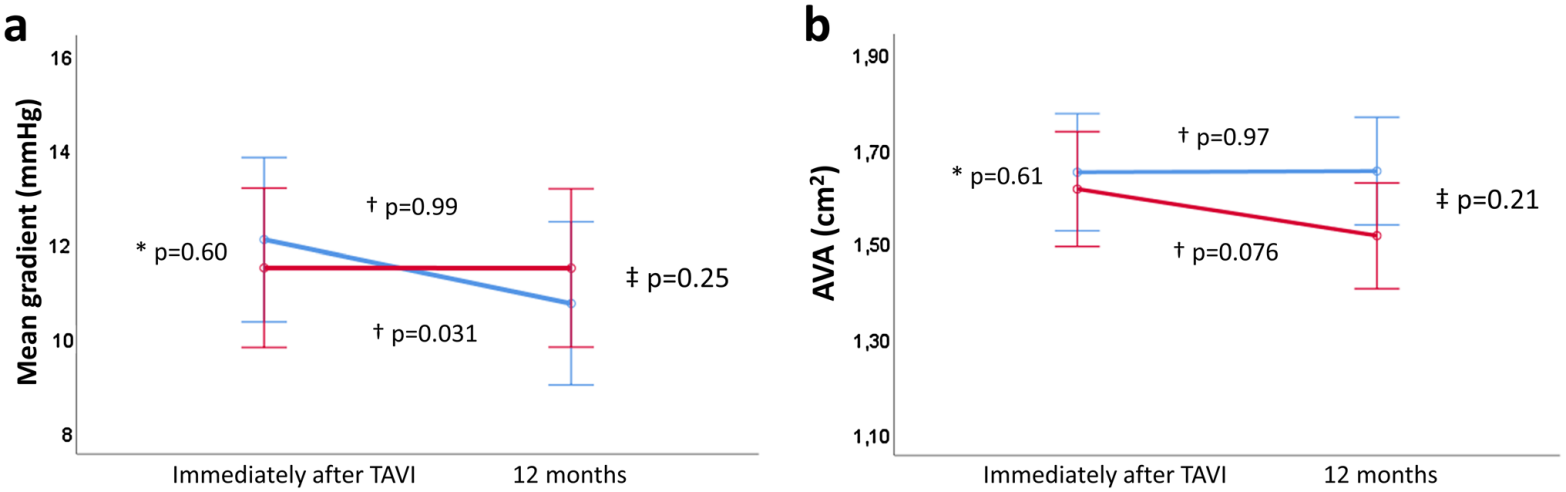
Prosthetic valve hemodynamics of patients with (red line) and without hypo-attenuated leaflet thickening (blue line) after transcatheter aortic valve implantation. Changes in transvalvular mean gradient (**a**) and prosthetic aortic valve area (AVA, **b**) from baseline to 12 months follow-up after transcatheter aortic valve implantation. *shows p value for comparing means between groups at baseline. †shows p value for groups over time. ‡shows p for interaction between groups over time

Transvalvular mean gradient remained unchanged in patients with HALT over time (from 11.4 ± 6.2 to 11.4 ± 7.1 mmHg, p = 0.997), but showed a slight decrease in patients without HALT (from 12.1 ± 5.9 to 10.8 ± 4.4 mmHg, p = 0.031). However, there were no statistically significant differences between groups over time (p for interaction = 0.25); but the decrease in mean gradient in patients without HALT during follow-up was significantly different if corrected for oral anticoagulation treatment (adjusted p for interaction = 0.049). Patients with HALT showed a trend towards smaller AVA at 12 months follow-up (from 1.61 ± 0.42 to 1.51 ± 0.38 cm^2^, p = 0.076), whereas AVA remained unchanged in patients without HALT (from 1.67 ± 0.44 to 1.66 ± 0.44 cm^2^, p = 0.97). However, no statistically significant differences were observed between the groups over time (p for interaction = 0.21, adjusted p for interaction = 0.064). Figure 3 illustrates changes in prosthetic valve hemodynamics (AVA, transvalvular gradient) between patients with and without HALT over time.

At 12 months follow-up echocardiography, seven patients showed an increased transvalvular mean gradient (Table 4). Four of these patients manifested immediately after TAVI and 3 patients (all with HALT) developed increased transvalvular gradients during follow-up. Of these 3 patients, one developed a significantly increased gradient of 44 mmHg; however, the transvalvular gradients of the two other patients were only slightly elevated (from 17 to 20 and 23 mmHg, respectively). Abnormal valve hemodynamics indicating prosthetic valve stenosis was present in 2 patients before discharge (1 patient with HALT and 1 without) and in 4 patients at 12 months follow-up (HALT: n = 3 (6%); no HALT: n = 1 (2%), p = 0.62). Whilst LVEF in the entire patient cohort improved significantly at 12 months follow-up, 21 patients (20%) showed a reduction in LVEF ≥ 5%; nevertheless, the prevalence of LVEF deterioration did not differ between patients with and without HALT (HALT: n = 9 (17%) versus no HALT: n = 12 (23%), p = 0.47). Moreover, absence of a reduction in LV volumes was observed in 24% of patients (HALT: n = 13 (25%) versus no HALT: n = 12 (23%), p = 0.82), whereas the absence of LV mass regression was noted in 27% of patients (HALT: n = 13 (26%) versus no HALT: n = 15 (29%), p = 0.70) without differences between patients with and without HALT.

**Table 4:** Echocardiographic endpoints after TAVI at 12 months follow-up

Variable	Overall population (n = 106)	HALT (n = 53)	no HALT (n = 53)	p value
Mean transvalvular gradient ≥ 20 mmHg, n (%)	7 (7)	5 (10)	2 (4)	0.44
Mean gradient, mmHg	11.1 ± 5.9	11.4 ± 7.1	10.8 ± 4.4	0.59
AVA ≤ 1.1 cm^2^, n (%)	14 (14)	9 (18)	5 (10)	0.25
AVA, cm^2^	1.59 ± 0.40	1.51 ± 0.38	1.66 ± 0.44	0.054
Possible THV obstruction, n (%) *	4 (4)	3 (6)	1 (2)	0.62
Decrease in LVEF ≥ 5%, n (%)	21 (20)	9 (17)	12 (23)	0.47
No reduction in LVEDV or LVESV, n (%)	25 (24)	13 (25)	12 (23)	0.82
No reduction in LV mass, n (%)	28 (27)	13 (26)	15 (29)	0.70

Data are presented as mean ± SD and n (%)

*Defined as a MG ≥ 20 mmHg and AVA ≤ 1.1 cm^2^

*AVA* aortic valve area, *LVEDV* left ventricular end-diastolic volume, *LVEF* left ventricular ejection fraction, *LVESV* left ventricular end-systolic volume, *TAVI* transcatheter aortic valve implantation, *THV* transcatheter heart valve

### Clinical outcomes after TAVI

Clinical outcomes are reported in Table 5. During a median follow-up of 1.0 year (IQR 1.0–2.3 years), 2 patients had a stroke/TIA (both patients without HALT, with 1 event being immediately after TAVI); 11 patients were admitted to the hospital because of heart failure (HALT: n = 3 (6%) versus no HALT: n = 8 (15%), log-rank χ^2^: 2.019, p = 0.079); 12 patients developed new-onset atrial fibrillation (HALT: n = 7 (15%) versus no HALT: n = 5 (10%), log-rank χ^2^: 0.440, p = 0.51) and 31 patients died (HALT: n = 15 (28%) versus no HALT: n = 16 (30%), log-rank χ^2^: 0.298, p = 0.59). No differences in event rates were observed between groups. After the diagnosis of HALT, 23 patients (43%) received medical treatment with oral anticoagulation, 13 patients (25%) already used oral anticoagulation because of atrial fibrillation, and medical therapy was not changed in 17 patients (32%).

**Table 5:** Clinical outcomes after TAVI

Variable	Overall population	HALT	No halt	p value*
Stroke/TIA	2 (2)	0	2 (4)	0.16
Heart failure hospitalizations	11 (10)	3 (6)	8 (15)	0.079
New-onset atrial fibrillation	12 (12)	7 (15)	5 (10)	0.51
All-cause mortality	31 (29)	15 (28)	16 (30)	0.59

Data are presented as n (%)

*TIA* transient ischemic attack

*p value comparing the event-free survival of clinical outcomes between patients with and without HALT using the log-rank test

## Discussion

The main findings of the current study can be summarized as follows: (1) Patients with and without HALT showed a similar reduction in LV volumes, regression in LV mass, and improvement in LVEF at 12 months after TAVI; (2) Prosthetic valve hemodynamics were comparable between groups over time; (3) The number of clinical events after TAVI was low and not significantly different between patients with and without HALT.

### Clinical consequences of HALT

HALT of transcatheter aortic valves is not uncommon. On MDCT, HALT is visible as hypo-attenuated areas at the aortic side of the transcatheter valve leaflet and is considered to reflect leaflet thrombosis [12, 29]. In the current case–control study, patients with and without HALT were compared.

The clinical impact of HALT ranges from an incidental finding on MDCT without effect on prosthetic valve hemodynamics and without clinical events, to manifest symptomatic transcatheter valve thrombosis. With the increasing use of TAVI to younger patients with lower surgical risk, it is essential to understand the clinical consequences of HALT. However, since the first publication of HALT by Pache et al. in 2013 [19], its clinical implications have been a subject of debate.

The clinical consequences associated with HALT can be classified into symptoms due to transcatheter valve obstruction, abnormal prosthetic valve hemodynamics on echocardiography, and clinical events. However, the presence of HALT is not associated with symptoms in most patients.

Overt valve thrombosis leads to transcatheter valve obstruction with subsequent symptoms. Two studies reported (worsening of) dyspnea in 38.9% of patients and 65.8% of them had clinical valve thrombosis [30, 31]. However, in a multicenter registry evaluating the echocardiographic predictors of HALT in low-risk patients undergoing TAVI, Khan et al. reported no differences in 6-min walking distance between patients with and without HALT at 30-day and 1-year follow-up [18]. Moreover, none of the 27 patients with HALT presented with clinical signs of heart failure or exertional dyspnea [18].

The effect of HALT on prosthetic valve hemodynamics has been evaluated in various studies. Data from two prospective registries reported that the mean aortic valve gradient was greater in patients with HALT and a mean transvalvular gradient ≥ 20 mmHg was more frequently observed [32]. In addition, the PARTNER 3 CT sub-study reported a trend towards a higher mean transvalvular gradient in patients with HALT [33]. Khan et al. reported worse valve hemodynamics (reduced AVA and Doppler velocity index) in patients with HALT at 30 days after TAVI, which had normalized at 1-year follow-up [18]. Moreover, results from a multicenter registry reported by Yanagisawa et al. showed similar mean transvalvular gradients in patients with versus without HALT at 1 and 2 years follow-up [21]. Various other studies showed no statistical differences in transvalvular gradients on echocardiography in patients with versus without HALT [16, 20, 34]. Our findings are in line with these results: a slight but significant decrease in gradient after TAVI was noted in patients without HALT (from 12.1 ± 5.9 mmHg immediately after TAVI to 10.8 ± 4.4 mmHg at 12 months follow-up, p = 0.031), while the transvalvular mean gradient was not elevated and remained unchanged over time in all patients with HALT (from 11.4 ± 6.2 mmHg immediately after TAVI to 11.4 ± 7.1 mmHg at 12 months follow-up, p = 0.997). Importantly, there were no statistically significant differences in transvalvular gradients between patients with versus without HALT over time.

Clinical outcomes associated with HALT include primarily adverse cerebrovascular events and mortality. Thromboembolic complications (stroke or TIA) were most commonly reported. However, the overall incidence of events is low and differences in clinical outcomes between patients with HALT and those without are absent. One study by Chakravarty et al. reported a significantly higher incidence of post-procedural stroke or TIA in the group of patients with reduced leaflet motion (associated with valve thrombosis) versus patients without [32]. In contrast, several studies reported no differences in stroke or TIA and other clinical outcomes between patients with and without HALT [21, 33, 34]. Moreover, in a recently published meta-analysis investigating the association of subclinical leaflet thrombosis with ischemic cerebral events and mortality, Casula et al. reported that subclinical leaflet thrombosis was not associated with a significant increase in cerebrovascular events and all-cause mortality after TAVI [35]. Previously, Vollema et al. reported that neither HALT nor increased transvalvular gradient was associated with stroke or TIA [13]. Similarly, in the current study, HALT was not associated with stroke/TIA, new-onset atrial fibrillation, heart failure hospitalization, or death.

### LV reverse remodeling

LV reverse remodeling is considered to be a beneficial process following LV afterload reduction after aortic valve replacement and has been associated with improved long-term outcomes [9]. The current results demonstrate that LV reverse remodeling after TAVI is similar in patients with and without HALT. In this multicenter case–control study, patients with severe aortic stenosis treated with TAVI showed a significant reduction in LVEDVi and LVESVi with an improvement in LVEF, accompanied by a reduction in LV mass at 12 months follow-up.

We hypothesized that the hemodynamic consequences of HALT might impair LV reverse remodeling after TAVI. However, our study demonstrated that HALT was not associated with increased prosthetic valve gradients, and therefore, may had no impact on LV reverse remodeling. Yet, some patients have been treated with antithrombotic therapy after the diagnosis of HALT, which might have prevented progression of HALT and potentially subsequent deterioration of prosthetic valve function ^32^. However, we found no differences in LV reverse remodeling between patients with and without HALT after adjusting for oral anticoagulation treatment. Our findings suggest that HALT, as it emerges in current clinical practice, seems to have limited clinical impact and may not lead to increased prosthetic gradients, impaired LV reverse remodeling, and worse outcome at 1-year follow-up after TAVI.

One other study evaluated the relation between HALT and LV reverse remodeling. Szilveszter et al. performed MDCT in 117 patients after TAVI and showed HALT in 25.6% of patients [36]. The authors showed (similar to our findings) significant LV reverse remodeling after TAVI, with a reduction in LV mass. Conversely, they demonstrated an inverse relation between HALT and LV reverse remodeling: HALT was more prevalent in patients with less than 20% reduction in LV mass at follow-up. This difference between the studies could be related to differences in imaging modality to assess LV mass (MDCT versus echo) and the timing of assessing LV mass after TAVI (3 months versus 12 months). Possibly, LV reverse remodeling is a process that may need more time and might occur up to 2 years after TAVI [10]. Finally, Szilveszter and colleagues [36] used a pre-defined threshold of 20% LV mass regression to define LV reverse remodeling, whereas in the current study LV mass regression was treated as a continuous variable. Additional studies are needed to further elucidate this issue.

The current study has some limitations. First, the presence of HALT was determined from MDCT ranging from 1 to 3 months after TAVI. Studies have reported the occurrence of HALT up to 3 years after TAVI [21, 33]. Second, only the presence of HALT was reported without providing detailed information about the extent of HALT. Last, no serial follow-up CT scans were performed; accordingly, no conclusions can be drawn regarding the extent and natural course of HALT.

## Conclusion

Improvement in LVEF and LV reverse remodeling at 12 months after TAVI were not limited by HALT. In addition, the number of clinical events was low and not different in patients with versus without HALT.

## Data Availability

The data underlying this article will be shared on reasonable request to the corresponding author.

## References

[1] Grossman W, Jones D, McLaurin LP (1975). Wall stress and patterns of hypertrophy in the human left ventricle. J Clin Invest.

[2] Otto CM, Prendergast B (2014). Aortic-valve stenosis–from patients at risk to severe valve obstruction. N Engl J Med.

[3] Orsinelli DA, Aurigemma GP, Battista S, Krendel S, Gaasch WH (1993). Left ventricular hypertrophy and mortality after aortic valve replacement for aortic stenosis. A high risk subgroup identified by preoperative relative wall thickness. J Am Coll Cardiol.

[4] Kang DH, Park SJ, Lee SA, Lee S, Kim DH, Kim HK, Yun SC, Hong GR, Song JM, Chung CH, Song JK, Lee JW, Park SW (2020). Early surgery or conservative care for asymptomatic aortic stenosis. N Engl J Med.

[5] Vollema EM, Singh GK, Prihadi EA, Regeer MV, Ewe SH, Ng ACT, Mertens BJA, Klautz RJM, Ajmone Marsan N, Bax JJ, Delgado V (2019). Time course of left ventricular remodelling and mechanics after aortic valve surgery: aortic stenosis vs. aortic regurgitation. Eur Heart J Cardiovasc Imaging.

[6] Kamperidis V, Joyce E, Debonnaire P, Katsanos S, van Rosendael PJ, van der Kley F, Sianos G, Bax JJ, Ajmone Marsan N, Delgado V (2014). Left ventricular functional recovery and remodeling in low-flow low-gradient severe aortic stenosis after transcatheter aortic valve implantation. J Am Soc Echocardiogr.

[7] Al-Hawwas M, Marmagkiolis K, Mehta JL (2017). The impact of transcatheter aortic valve implantation and surgical aortic valve replacement on left ventricular remodeling. Am J Cardiol.

[8] Kim SJ, Samad Z, Bloomfield GS, Douglas PS (2014). A critical review of hemodynamic changes and left ventricular remodeling after surgical aortic valve replacement and percutaneous aortic valve replacement. Am Heart J.

[9] Ali A, Patel A, Ali Z, Abu-Omar Y, Saeed A, Athanasiou T, Pepper J (2011). Enhanced left ventricular mass regression after aortic valve replacement in patients with aortic stenosis is associated with improved long-term survival. J Thorac Cardiovasc Surg.

[10] Une D, Mesana L, Chan V, Maklin M, Chan R, Masters RG, Mesana TG, Ruel M (2015). Clinical impact of changes in left ventricular function after aortic valve replacement: analysis from 3112 patients. Circulation.

[11] Lindman BR, Stewart WJ, Pibarot P, Hahn RT, Otto CM, Xu K, Devereux RB, Weissman NJ, Enriquez-Sarano M, Szeto WY, Makkar R, Miller DC, Lerakis S, Kapadia S, Bowers B, Greason KL, McAndrew TC, Lei Y, Leon MB, Douglas PS (2014). Early regression of severe left ventricular hypertrophy after transcatheter aortic valve replacement is associated with decreased hospitalizations. J Am Coll Cardiol Intv.

[12] Ng ACT, Holmes DR, Mack MJ, Delgado V, Makkar R, Blanke P, Leipsic JA, Leon MB, Bax JJ (2020). Leaflet immobility and thrombosis in transcatheter aortic valve replacement. Eur Heart J.

[13] Vollema EM, Kong WKF, Katsanos S, Kamperidis V, van Rosendael PJ, van der Kley F, de Weger A, Ajmone Marsan N, Delgado V, Bax JJ (2017). Transcatheter aortic valve thrombosis: the relation between hypo-attenuated leaflet thickening, abnormal valve haemodynamics, and stroke. Eur Heart J.

[14] Makkar RR, Fontana G, Jilaihawi H, Chakravarty T, Kofoed KF, De Backer O, Asch FM, Ruiz CE, Olsen NT, Trento A, Friedman J, Berman D, Cheng W, Kashif M, Jelnin V, Kliger CA, Guo H, Pichard AD, Weissman NJ, Kapadia S, Manasse E, Bhatt DL, Leon MB, Sondergaard L (2015). Possible subclinical leaflet thrombosis in bioprosthetic aortic valves. N Engl J Med.

[15] Hansson NC, Grove EL, Andersen HR, Leipsic J, Mathiassen ON, Jensen JM, Jensen KT, Blanke P, Leetmaa T, Tang M, Krusell LR, Klaaborg KE, Christiansen EH, Terp K, Terkelsen CJ, Poulsen SH, Webb J, Botker HE, Norgaard BL (2016). Transcatheter aortic valve thrombosis: incidence, predisposing factors, and clinical implications. J Am Coll Cardiol.

[16] Sondergaard L, De Backer O, Kofoed KF, Jilaihawi H, Fuchs A, Chakravarty T, Kashif M, Kazuno Y, Kawamori H, Maeno Y, Bieliauskas G, Guo H, Stone GW, Makkar R (2017). Natural history of subclinical leaflet thrombosis affecting motion in bioprosthetic aortic valves. Eur Heart J.

[17] Rashid HN, Brown AJ, McCormick LM, Amiruddin AS, Be KK, Cameron JD, Nasis A, Gooley RP (2018). Subclinical leaflet thrombosis in transcatheter aortic valve replacement detected by multidetector computed tomography- a review of current evidence. Circ J.

[18] Khan JM, Rogers T, Waksman R, Torguson R, Weissman G, Medvedofsky D, Craig PE, Zhang C, Gordon P, Ehsan A, Wilson SR, Goncalves J, Levitt R, Hahn C, Parikh P, Bilfinger T, Butzel D, Buchanan S, Hanna N, Garrett R, Shults C, Garcia-Garcia HM, Kolm P, Satler LF, Buchbinder M, Ben-Dor I, Asch FM (2019). Hemodynamics and subclinical leaflet thrombosis in low-risk patients undergoing transcatheter aortic valve replacement. Circ Cardiovasc Imaging.

[19] Pache G, Schoechlin S, Blanke P, Dorfs S, Jander N, Arepalli CD, Gick M, Buettner HJ, Leipsic J, Langer M, Neumann FJ, Ruile P (2016). Early hypo-attenuated leaflet thickening in balloon-expandable transcatheter aortic heart valves. Eur Heart J.

[20] Tang L, Lesser JR, Schneider LM, Burns MR, Gossl M, Garberich R, Niikura H, Witt D, Sorajja P (2019). Prospective evaluation for hypoattenuated leaflet thickening following transcatheter aortic valve implantation. Am J Cardiol.

[21] Yanagisawa R, Tanaka M, Yashima F, Arai T, Jinzaki M, Shimizu H, Fukuda K, Watanabe Y, Naganuma T, Higashimori A, Mizutani K, Araki M, Tada N, Yamanaka F, Otsuka T, Yamamoto M, Hayashida K (2019). Early and late leaflet thrombosis after transcatheter aortic valve replacement. Circ Cardiovasc Interv.

[22] Del Trigo M, Munoz-Garcia AJ, Wijeysundera HC, Nombela-Franco L, Cheema AN, Gutierrez E, Serra V, Kefer J, Amat-Santos IJ, Benitez LM, Mewa J, Jimenez-Quevedo P, Alnasser S, Garcia Del Blanco B, Dager A, Abdul-Jawad Altisent O, Puri R, Campelo-Parada F, Dahou A, Paradis JM, Dumont E, Pibarot P, Rodes-Cabau J (2016). Incidence, timing, and predictors of valve hemodynamic deterioration after transcatheter aortic valve replacement: multicenter registry. J Am Coll Cardiol.

[23] Issa IF, Dahl JS, Poulsen SH, Waziri F, Pedersen CT, Riber L, Sogaard P, Moller JE (2020). The relation of structural valve deterioration to adverse remodelling and outcome in patients with biological heart valve prostheses. Eur Heart J Cardiovasc Imaging.

[24] Blanke P, Weir-McCall JR, Achenbach S, Delgado V, Hausleiter J, Jilaihawi H, Marwan M, Norgaard BL, Piazza N, Schoenhagen P, Leipsic JA (2019). Computed tomography imaging in the context of transcatheter aortic valve implantation (TAVI)/transcatheter aortic valve replacement (TAVR): an expert consensus document of the society of cardiovascular computed tomography. J Am Coll Cardiol Img.

[25] Otto CM, Kumbhani DJ, Alexander KP, Calhoon JH, Desai MY, Kaul S, Lee JC, Ruiz CE, Vassileva CM (2017). 2017 ACC expert consensus decision pathway for transcatheter aortic valve replacement in the management of adults with aortic stenosis: a report of the american college of cardiology task force on clinical expert consensus documents. J Am Coll Cardiol.

[26] Lang RM, Badano LP, Mor-Avi V, Afilalo J, Armstrong A, Ernande L, Flachskampf FA, Foster E, Goldstein SA, Kuznetsova T, Lancellotti P, Muraru D, Picard MH, Rietzschel ER, Rudski L, Spencer KT, Tsang W, Voigt JU (2015). Recommendations for cardiac chamber quantification by echocardiography in adults: an update from the American Society of Echocardiography and the European Association of Cardiovascular Imaging. J Am Soc Echocardiogr.

[27] Lancellotti P, Pibarot P, Chambers J, Edvardsen T, Delgado V, Dulgheru R, Pepi M, Cosyns B, Dweck MR, Garbi M, Magne J, Nieman K, Rosenhek R, Bernard A, Lowenstein J, Vieira ML, Rabischoffsky A, Vyhmeister RH, Zhou X, Zhang Y, Zamorano JL, Habib G (2016). Recommendations for the imaging assessment of prosthetic heart valves: a report from the European Association of Cardiovascular Imaging endorsed by the Chinese Society of Echocardiography, the Inter-American Society of Echocardiography, and the Brazilian Department of Cardiovascular Imaging. Eur Heart J Cardiovasc Imaging.

[28] Annoni AD, Andreini D, Pontone G, Mancini ME, Formenti A, Mushtaq S, Baggiano A, Conte E, Guglielmo M, Muscogiuri G, Muratori M, Fusini L, Trabattoni D, Teruzzi G, Coutinho Santos AI, Agrifoglio M, Pepi M (2018). CT angiography prior to TAVI procedure using third-generation scanner with wide volume coverage: feasibility, renal safety and diagnostic accuracy for coronary tree. Br J Radiol.

[29] Sellers SL, Turner CT, Sathananthan J, Cartlidge TRG, Sin F, Bouchareb R, Mooney J, Norgaard BL, Bax JJ, Bernatchez PN, Dweck MR, Granville DJ, Newby DE, Lauck S, Webb JG, Payne GW, Pibarot P, Blanke P, Seidman MA, Leipsic JA (2019). Transcatheter aortic heart valves: histological analysis providing insight to leaflet thickening and structural valve degeneration. J Am Coll Cardiol Img.

[30] Jose J, Sulimov DS, El-Mawardy M, Sato T, Allali A, Holy EW, Becker B, Landt M, Kebernik J, Schwarz B, Richardt G, Abdel-Wahab M (2017). Clinical bioprosthetic heart valve thrombosis after transcatheter aortic valve replacement: incidence, characteristics, and treatment outcomes. J Am Coll Cardiol Intv.

[31] Latib A, Naganuma T, Abdel-Wahab M, Danenberg H, Cota L, Barbanti M, Baumgartner H, Finkelstein A, Legrand V, de Lezo JS, Kefer J, Messika-Zeitoun D, Richardt G, Stabile E, Kaleschke G, Vahanian A, Laborde JC, Leon MB, Webb JG, Panoulas VF, Maisano F, Alfieri O, Colombo A (2015). Treatment and clinical outcomes of transcatheter heart valve thrombosis. Circ Cardiovasc Interv.

[32] Chakravarty T, Sondergaard L, Friedman J, De Backer O, Berman D, Kofoed KF, Jilaihawi H, Shiota T, Abramowitz Y, Jorgensen TH, Rami T, Israr S, Fontana G, de Knegt M, Fuchs A, Lyden P, Trento A, Bhatt DL, Leon MB, Makkar RR, Resolve and Investigators S (2017). Subclinical leaflet thrombosis in surgical and transcatheter bioprosthetic aortic valves: an observational study. Lancet.

[33] Makkar RR, Blanke P, Leipsic J, Thourani V, Chakravarty T, Brown D, Trento A, Guyton R, Babaliaros V, Williams M, Jilaihawi H, Kodali S, George I, Lu M, McCabe JM, Friedman J, Smalling R, Wong SC, Yazdani S, Bhatt DL, Bax J, Kapadia S, Herrmann HC, Mack M, Leon MB (2020). Subclinical leaflet thrombosis in transcatheter and surgical bioprosthetic valves: PARTNER 3 cardiac computed tomography substudy. J Am Coll Cardiol.

[34] Ruile P, Minners J, Breitbart P, Schoechlin S, Gick M, Pache G, Neumann FJ, Hein M (2018). Medium-term follow-up of early leaflet thrombosis after transcatheter aortic valve replacement. J Am Coll Cardiol Intv.

[35] Casula M, Fortuni F, Ferlini M, Mauri S, Rebuffi C, Rossini R, Ferrario M, Visconti LO (2020). Subclinical leaflet thrombosis after transcatheter aortic valve replacement: a meaningless finding? A systematic review and meta-analysis. Eur Heart J Qual Care Clin Outcomes.

[36] Szilveszter B, Oren D, Molnar L, Apor A, Nagy AI, Molnar A, Vattay B, Kolossvary M, Karady J, Bartykowszki A, Jermendy AL, Suhai FI, Panajotu A, Maurovich-Horvat P, Merkely B (2019). Subclinical leaflet thrombosis is associated with impaired reverse remodelling after transcatheter aortic valve implantation. Eur Heart J Cardiovasc Imaging.

